# Predicting vegetative phase nutrient uptake in *Cannabis sativa* L. via transpiration-driven mass-balance

**DOI:** 10.3389/fpls.2025.1753553

**Published:** 2026-01-22

**Authors:** Kit Powell, William L. Bauerle

**Affiliations:** Department of Horticulture and Landscape Architecture, Colorado State University, Fort Collins, CO, United States

**Keywords:** element uptake, hemp, hydroponics, nutrient use, water use efficiency

## Abstract

The rapid expansion of *Cannabis sativa* L. cultivation necessitates fertilization strategies that optimize nutrient delivery and resource use. A transpiration-driven mass balance framework quantified nutrient removal from solution relative to whole-plant water use in two *Cannabis sativa* L. cultivars, CJ2 and First Light. Uptake measured via the pour-through method was compared against total and organ-specific tissue concentrations, verifying cultivar-specific mass balance nutrient uptake estimates. The objective was to formulate a nutrient replenishment strategy proportional to water uptake, enabling continuous, cultivar-specific fertilization optimization. Results revealed cultivar-specific uptake patterns: nitrogen ranged from 97–155 mg L^-1^ (First Light) and 78–145 mg L^-1^ (CJ2); phosphorus from 14–48 mg L^-1^ and 13–49 mg L^-1^; and potassium from 112–216 mg L^-1^ and 111–205 mg L^-1^, respectively. Water use efficiency (WUE) was similar across cultivars (CJ2: 4.71 g L^-1^ H_2_O; First Light: 4.59 g L^-1^ H_2_O). Temporal analysis normalized to transpired water showed peak nitrogen and potassium uptake during week 1, with significant declines by week 2 (p < 0.001), while calcium and magnesium uptake increased steadily (p < 0.0001). These findings support a scalable nutrient forecasting method based on nutrient uptake from solution that is proportional to whole-plant water use. Integrating transpiration data with tissue nutrient profiles enables cultivar-specific fertigation strategies that minimize waste and enhance sustainability in cannabis production.

## 1 Introduction

Despite rising fertilizer costs, nutrient delivery in hydroponic and containerized systems remains largely guided by empirically derived recipes rather than mechanistic understanding of elemental uptake. These formulations rarely reflect quantitative, element-specific plant demand. As a result, this often leads to nutrient over-application, increased production costs, and elevated nutrient concentrations in leachate (Bugbee, 2004; Sonneveld and Voogt, 2009; Langenfeld and Bugbee, 2024). In addition, fixed recipes fail to account for dynamic changes in nutrient demand across genotype and developmental stages. This limitation can produce inaccurate elemental application and reduced sustainability (Marschner, 2012; Caplan et al., 2018; Bernstein et al., 2019; Saloner and Bernstein, 2021; Langenfeld et al., 2022).

Given the economic and regulatory importance of *Cannabis sativa* L. (*C. sativa*) production, there is a critical need for nutrient management strategies that align with physiological demand while minimizing environmental impact (Bernstein et al., 2019; Saloner and Bernstein, 2021). Mass balance approaches, grounded in the principle of elemental conservation, provide a quantitative framework for assessing nutrient uptake. Specifically, nutrient uptake is estimated as the difference between known nutrient inputs and measurable elemental accumulation in plant tissues (Silberbush and Ben-Asher, 2001; Bugbee, 2004; Langenfeld et al., 2022). When combined with measurements of nutrient depletion from the root-zone solution and organ-specific tissue concentrations, this framework allows total elemental uptake and intra-plant organ allocation to be quantified (Bugbee, 2004; White and Brown, 2010; Saloner and Bernstein, 2020). Although mass balance methods have been applied in several other crops, intra-plant organ allocation has not been examined in *C. sativa.* This gap reflects, in part, the analytical demands associated with destructive sampling, tissue analysis, and precise fertigation monitoring (Shiponi and Bernstein, 2021; Konvalina et al., 2024). Accordingly, the objective of this study was to evaluate organ-specific and organ biomass-weighted mass balance frameworks for quantifying whole-plant nutrient uptake in high-value *C. sativa* production systems.

Plant acquisition of essential mineral elements occurs primarily from the rhizosphere (Cui, 2012; White and Brown, 2010), a process that can be quantified using mass balance principles to account for nutrient inputs and whole-plant storage (Cogley, 2010; Langenfeld et al., 2022). In controlled-environment systems, closed-loop fertigation supports steady-state nutrition, whereby water lost to transpiration is replaced with nutrient solution supplying elements taken up with that water (Langenfeld and Bugbee, 2024), thereby linking nutrient delivery to transpiration demand and improving nutrient-use efficiency (NUE). Within this framework, Langenfeld et al. (2022) computed irrigation solution element concentrations as the product of target leaf tissue concentrations and whole-plant water-use efficiency (WUE), yielding nutrient input concentrations (mg L^-1^). Building on this approach, we apply an organ-explicit, biomass-weighted mass balance framework that links nutrient input calculations to developmental-stage uptake and transpiration-driven water use and facilitates comparison between solution nutrient depletion with whole-plant nutrient uptake, which we evaluate using pour-through leachate analysis (Altland, 2021).

This study evaluated whether nutrient depletion from the fertigation solution corresponds to cumulative nutrient accumulation in discrete plant organs or whether organ biomass-weighted accumulation more precisely reflects whole-plant nutrient uptake in relation to WUE dynamics. To address this question, we pursued four specific objectives. First, we quantified whole-plant nutrient uptake from solution depletion using pour-through leachate analysis. Second, we determined organ-specific element accumulation via ICP-OES. Third, we tracked cumulative water use and calculated whole-plant WUE. Fourth, we evaluated agreement between nutrient extraction from solution and both organ-explicit and biomass-weighted tissue concentration × WUE calculations used for fertigation recipe formulation. To our knowledge, this is the first study to directly compare organ-explicit and organ biomass-weighted mass balance frameworks in *C. sativa* under controlled environment conditions. This work aims to advance resource-use efficiency in *C. sativa* production and to inform broader controlled-environment agriculture systems in which nutrient stewardship is essential for optimizing resource use and sustainability.

## 2 Materials and methods

### 2.1 Plant material and clonal propagation

This study evaluated the WUE and nutrient uptake dynamics of *C. sativa* cultivars ‘CJ2’ and ‘First Light’ using a mass balance approach. Lateral branch segments, 20 cm in length, were taken from mother plants for clonal propagation. On June 14, 2024, 1-inch rockwool cubes (Growdan, Milton, Ontario, Canada) were presoaked in deionized water (DI) containing a diluted complete fertilizer solution (17-5–17 Cal-Mag Special, Plant Marvel, Chicago Heights, Illinois, USA) at an electrical conductivity (EC) of 0.3 dS·m^-1^ adjusted to pH 6.0.

Each cutting was prepared by making a diagonal incision at the basal end with lower leaves removed leaving two nodes, followed by immersion in a rooting gel containing 3.3 g/L indole-3-butyric acid (Clonex, Growth Technology Ltd., Taunton, Somerset, UK). Cuttings were then inserted into the rockwool cubes. To minimize transpiration during root development, the leaves of each cutting were trimmed to approximately half their original surface area. A total of 16 cuttings (8 per cultivar) were propagated under a humidity dome on a 1020 horticultural tray. The tray was situated on a thermostatically controlled heat mat (Vivosun, 10” × 20.75”, Ontario, California, USA) and illuminated continuously (24 h photoperiod) by a germination light fixture (Hydrofarm Jump Start T5, 61cm, Shoemakersville, Pennsylvania, USA). Daily misting with DI and periodic reapplication of 0.3 dS·m^-1^ fertilizer solution to the rockwool cube occurred until root development.

After 12 days, visible roots emerged from the rockwool cubes. The rooted cuttings were transplanted into 10 cm rockwool cubes presoaked in a complete fertilizer solution (17-5–17 Cal-Mag Special, Plant Marvel) with an EC of 1.0 mS/cm, and pH of 6.0. Plants were then transferred to a growth chamber (Conviron, Model PG2500, Pembina, North Dakota, USA) and maintained under controlled conditions: 24-h photoperiod with a photosynthetic photon flux density (PPFD) of 340 µmol·m^-2^·s^-1^, air temperature of 22°C, and 40% RH. Over the following 10 days, chamber conditions were progressively increased to match those of the research greenhouse with an air temperature setpoint of 26.5°C and liquid feed provided to the plants at an EC of 1.0 dS·m^-1^ and pH of 6.0.

### 2.2 Greenhouse experimental design and irrigation

Following environmental acclimation, the plants were moved to a research greenhouse at the Colorado State University Horticulture Center on July 7, 2024. The environmental conditions of the bay were adjusted via venting, forced air, and an end wall evaporative cooler to a set point of 26.5 °C, and natural gas heating set to initiate at 18.3°C. The vapor pressure deficit (VPD) varied over the experiment, with the majority of daily mean values between 1.2 and 1.6 kPa. Each cultivar was arranged in a single row of eight plants spaced 1.2 m between plants in the row and 1.5 m between the rows, totaling 16 plants. The rockwool cubes were transplanted into 11-L Bato buckets prefilled with 1.5 kg of dry horticultural-grade perlite, which was selected due to its inability to chemically react with nutrient ions in solution.

To periodically deliver essential elements to the growing substrate, two dripper stakes per container delivered irrigation at a rate of 1.9 liter·min^-1^. Irrigation occurred at four daily intervals (0700, 1100, 1400, and 1900 h) for 10 minutes per cycle. The schedule was subsequently changed to two daily events (0700 and 2000 h) to establish an adequate uptake duration that was uninterrupted by irrigation events. To minimize substrate surface evaporative water loss, the surface of each container was covered with white plastic. Supplemental lighting was set to an 18/6-h (day/night) photoperiod based on vegetative industry standards. The natural photoperiod at the time of the experiment’s initiation was 14 h and 32 min calculated by the NOAA Solar Calculator (National Oceanic and Atmospheric Administration, n.d.).

After 16 days of initial growth in the greenhouse environment, essential elements were delivered via calibrated pneumatic pistons. The piston-driven injection system operates via compressed air which actuates individual element calibrated pistons to deliver fluid at a pressure setpoint maintained at 40 psi above the water pressure within the irrigation line. This ensures positive-pressure differential injection, enabling precise delivery of concentrated elements directly into an inline manifold to formulate a pre-determined complete recipe (nitrogen [N] - (nitrate – 250 ppm, ammonium - 6.3 ppm), potassium [K] – 350 ppm, phosphorus [P] – 50 ppm, magnesium [Mg] – 61.7 ppm, calcium [Ca] – 100 ppm, iron [Fe] – 2 ppm, zinc [Zn] – 0.23 ppm, boron [B] – 0.09 ppm, copper [Cu] – 0.23 ppm, molybdenum [Mo] – 0.07 ppm, manganese [Mn] – 0.27 ppm). The injection systems irrigation manifold was fitted upstream of a 113.5 L mixing tank, which supplied the 1.9 liter·min^-1^ downstream dripper stakes with a complete nutrient solution.

### 2.3 Gravimetric water use measurements and leachate sampling

Gravimetric balances (Adam Equipment GBK-35A, Oxford, Connecticut, USA) were positioned under four representative plants per cultivar, in row positions 1, 3, 5, and 7 for ‘CJ2’, and 2, 4, 6, and 8 for ‘First Light’. Each balance was connected to a custom-built IoT data acquisition system. The system consisted of a microcontroller (Adafruit ESP32 Feather, Brooklyn, New York, USA) powered by a lithium battery. The balance interfaces with data acquisition via an RS232 connection. Mass data were read and logged every 10 minutes via wireless transmission to a ThingSpeak dashboard (MathWorks, n.d.) for real-time monitoring and data storage.

Prior to substrate leachate extraction, substrate was watered to container capacity and allowed to drain for 30 minutes. Then, water transpired, measured continuously per container via gravimetric mass loss, was recorded throughout a 12 h day course. After 12 h of nutrient solution uptake, the quantity of solution transpired was replaced with DI to return the substrate to container capacity. Resupplied DI was permitted to stabilize for 25 minutes. Thereafter, an additional mass of DI displacement solution was added to the substrate surface to hydraulically push 230 ml of solution out of the drainage elbow. During drainage, samples were filtered through 18×16 mesh screen, and the first 230 ml of the solution was collected in clean, unused bottles. We note that the volume of displacement solution collected (approximately 2% of container volume) was predetermined to obtain a representative sample of the bulk substrate rather than the bottom layer alone, given that leachate measurements were intended solely as an internal consistency check for comparing nutrient uptake with supplied inputs (Altland, 2021). Samples were immediately transferred to refrigerated storage for later analysis. Samples were collected every third day to ensure the substrate returned to base recipe stabilization prior to repeated extraction. A total of ten leachate sampling events occurred throughout the 38-day vegetative growth phase (July 7 – August 13, 2024).

### 2.4 Plant measurements

Non-destructive individual plant biometric measurements of gravimetrically monitored plants were collected weekly. Stem caliper 5 cm above the rockwool surface was recorded as the average of two x and y direction caliper readings (mm). The height of the main stem was recorded as the distance from the surface edge of the Bato to the plant’s terminal tip. Measurement of crown horizontal expansion was recorded as the average distance from the main stem in two perpendicular directions.

### 2.5 Biomass and organ tissue analysis

After 38 days of vegetative growth monitoring, plants were destructively harvested. Immediately upon harvest, leaf area was quantified using a leaf area meter (LI-COR LI-3100, Lincoln, NE, USA). Root systems were carefully washed to remove perlite, then individual plant organs (leaves, stems, and roots) were dried at 70°C for ≥ five days until constant mass. Dry mass per organ was recorded, and organ specific tissue was homogenized using a masticating grinder to obtain replicate plant organ tissue samples of approximately 5 g (n = 4) per cultivar. Organ specific tissue element analysis occurred via Thermo iCAP PRO EPS Duo ICP-OES and NO_3_^−^ was quantified by flow injection analyzer (FIA; FIAlyser-2000, FIAlab) (Brookside Laboratories, New Breman, Ohio, USA).

#### 2.5.1 Nutrient solution input concentration estimation

Mass balance estimates of the individual element concentration required in nutrient solution were derived as described in Langenfeld et al. (2022). The predicted nutrient input concentration is the product of tissue element concentration (Equation 1) and plant WUE (Equation 2) where:

(1) $$C_{input}=C_{tissue}\;x\;WUE$$

Mean tissue nutrient concentration ( $C_{tissue}$*)* per organ was determined via ICP-OES analysis and used in combination with WUE to estimate individual element concentrations ( $C_{input}$) within the nutrient solution.

WUE was calculated as:

(2) $$WUE=\;\frac{M_{Dry}}{V_{irrigation}}$$

where ( $M_{Dry}$) is total plant dry biomass (g plant^-1^) and ( $V_{irrigation}$) is the total irrigation volume transpired per plant (L plant^-1^).

To estimate the whole-plant nutrient concentration, an organ biomass-weighted mean was calculated by its fractional contribution to the total biomass and then summed across all organs using the following equation:

(3) $$C_{weighted}=\sum_{i=1}^{n}(C_{organ}\;X\;\frac{Biomass_{organ}}{Biomass_{total}})$$

where ( $C_{weighted}$) is the whole-plant biomass-weighted mean, ( $C_{organ}$) represents the nutrient concentration measured in each individual organ (leaf, stem, root, or flower), ( $Biomass_{organ}$) is the dry biomass of the organ, and ( $Biomass_{total}$) is the total above and below-ground plant biomass. This approach integrates organ-specific nutrient concentrations with their proportional contribution to total biomass, providing a physiologically meaningful estimate of the average whole-plant concentration. In effect, organs with greater biomass exert more influence on the weighted value, allowing the calculation to more accurately represent the nutrient status of the entire plant rather than relying on a single tissue type.

### 2.6 Statistical analysis

Elemental uptake data were analyzed using repeated measures analysis of variance (RM-ANOVA), with cultivar as a between-subjects factor and date as a within-subjects factor. Data were organized by element, and each element was analyzed independently. The models were implemented using the ezANOVA function from the ez package in R (R Core Team, 2024, version 4.3.1), specifying replicate as the subject identifier to account for repeated observations. All categorical variables were converted to factors prior to analysis.

For each element, *post hoc* pairwise comparisons were conducted using estimated marginal means with the emmeans package. Bonferroni adjustment was applied to control the familywise error rate. Summary statistics, including means and standard errors, were computed for each cultivar × date combination and used to construct interaction plots.

Model assumptions were evaluated using residual diagnostics, and potential influential observations were identified using Cook’s distance and leverage diagnostics from the base lm function. All statistical analyses were performed in R (R Core Team, 2024, version 4.3.1), with significance determined at the α = 0.05 level.

Cultivar differences in tissue nutrient concentrations during the vegetative phase were evaluated using Welch’s two-sample *t*-tests implemented in R (R Core Team, 2024, version 4.3.1). Analyses were conducted separately for each element and tissue (leaf, stem, root, and biomass-weighted mean), using replicate plants (*n* = 4 per cultivar) as the experimental unit. Welch’s test was selected to accommodate unequal variances and small sample sizes. Mean values and standard errors were calculated from replicate-level observations, and statistical significance was assessed using two-tailed tests with an α level of 0.05. All analyses and figure generation were fully scripted to ensure reproducibility.

## 3 Results

**Figure 1:**
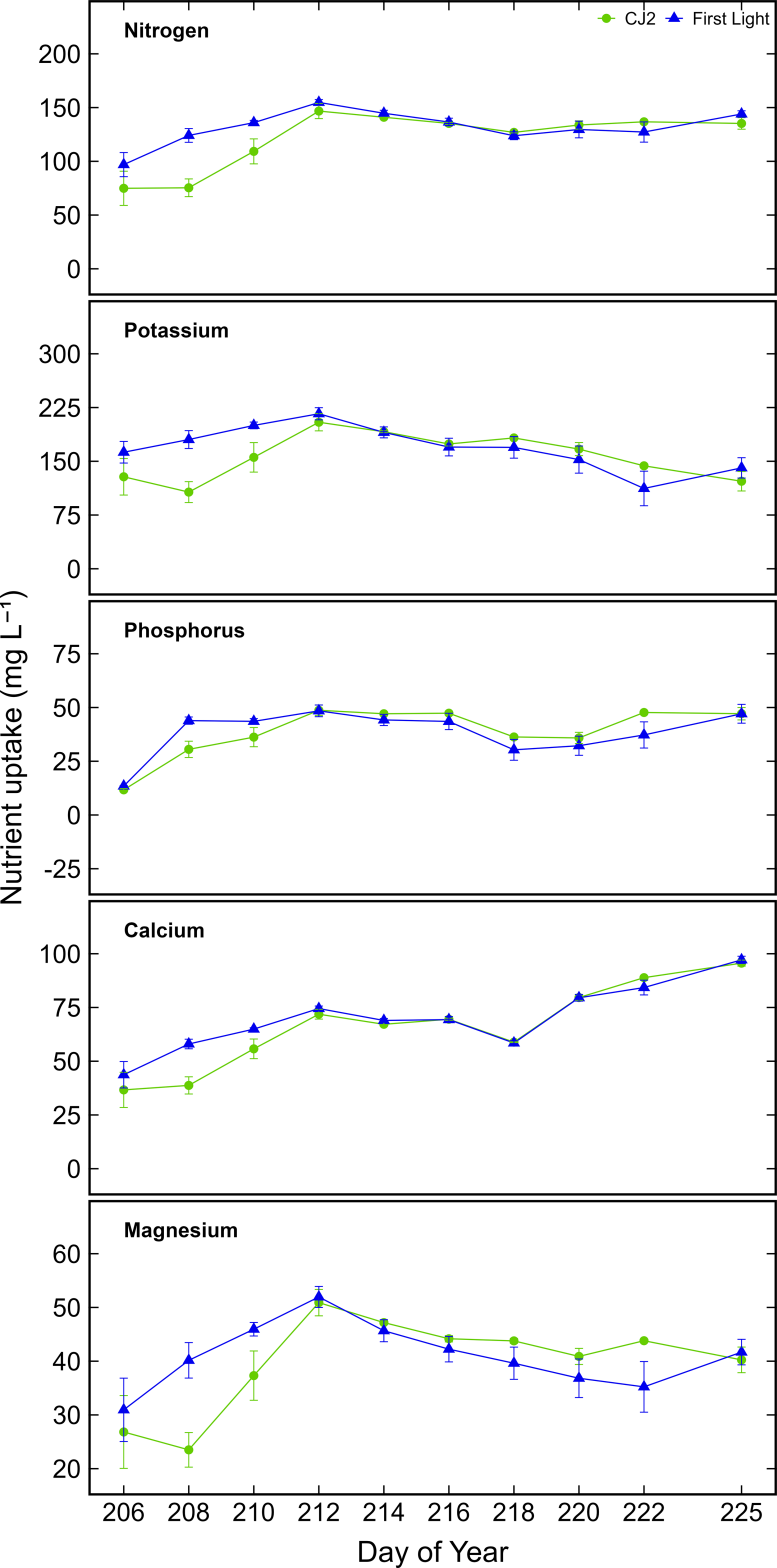
Calcium, magnesium, nitrogen, phosphorus, and potassium uptake from solution for cultivar CJ2 and First Light over ten repeated measure 12 h day uptake cycles over the course of the vegetative phase ± standard error of the mean. Data shown correspond to the active leachate sampling period following plant establishment.

**Figure 2:**
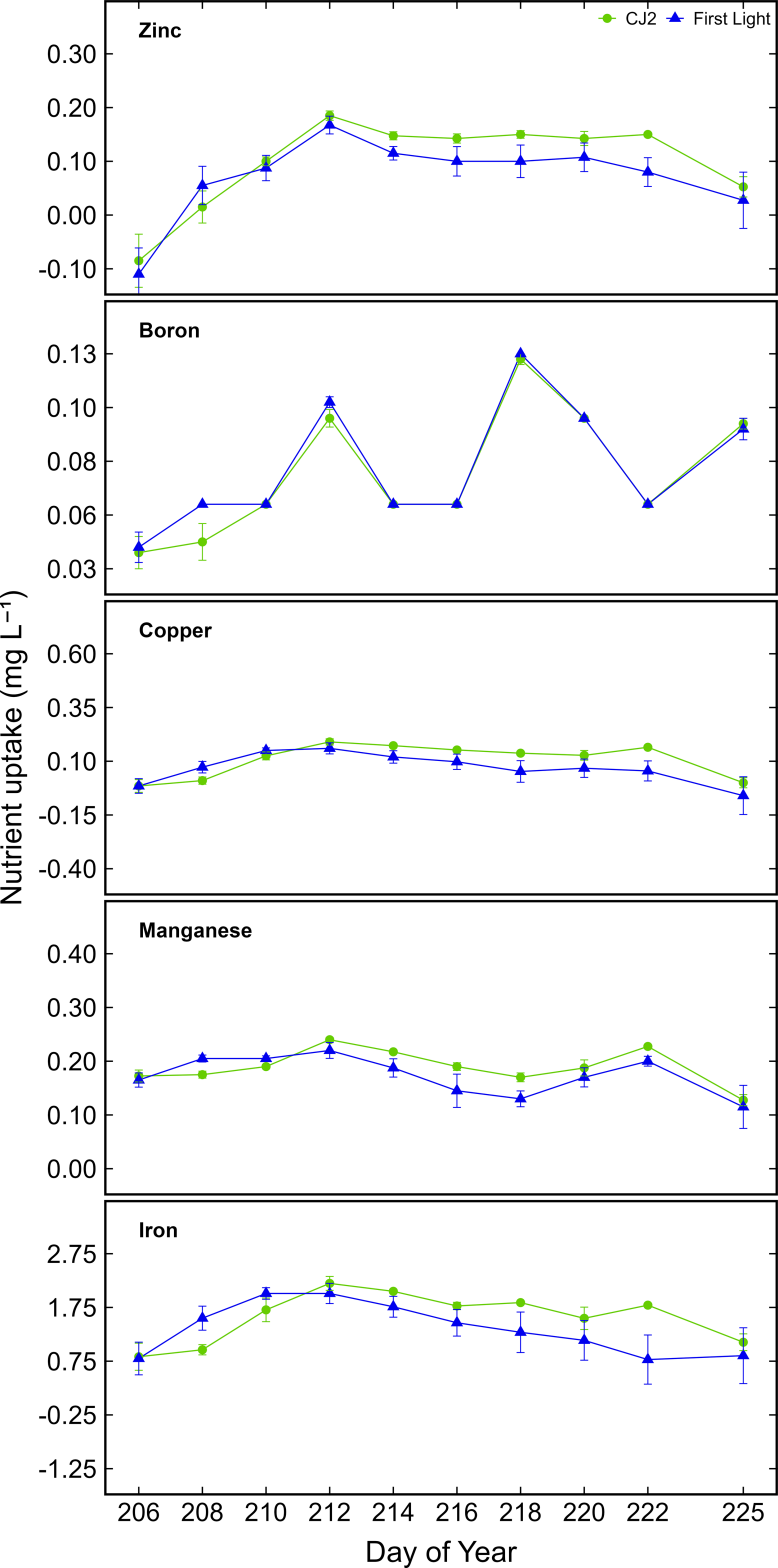
Boron, copper, iron, manganese, and zinc uptake from solution for cultivar CJ2 and First Light over ten repeated 12 h day uptake cycles over the course of the vegetative phase ± standard error of the mean. Data shown correspond to the active leachate sampling period following plant establishment.

Plants were clonally propagated beginning on 14 June 2024 and transferred to the research bay on 7 July 2024. Following transfer, a short establishment period was allowed to permit root systems to acclimate to the experimental containers and nutrient solution within the containers to stabilize before initiating leachate-based measurements. As a result, nutrient uptake and gravimetric water-use data collection commenced on 24 July 2024 and continued through 13 August 2024. **Figures 1** and **2** therefore depict the active measurement period (day of year 206–225), while the vegetative phase described here includes both the establishment and subsequent monitoring intervals.

**Figure 1** shows the cultivar difference in element uptake for N, K, P, Ca, and Mg. Cultivar-specific uptake patterns were present: N: ranged from 97–144 mg L^-1^ (First Light) and 75–135 mg L^-1^ (CJ2); K: from 112–216 mg L^-1^and 106–205 mg L^-1^; P: from 14–47 mg L^-1^ and 12–47 mg L^-1^; Ca: from 44–97 mg L^-1^and 37–96 mg L^-1^; and Mg: from 31–52 mg L^-1^ and 24–51 mg L^-1^, respectively. Moreover, the initial three leachate collections showed significant variation between cultivars, however, cultivar differences diminished thereafter (**Figure 1**). WUE was similar between cultivars (CJ2: 4.71 g L^-1^ H_2_O; First Light: 4.59 g L^-1^ H_2_O). Temporal analysis normalized to transpired water showed peak N and K uptake during week 1, with significant declines by week 2 (p < 0.001), while Ca and Mg uptake increased steadily over time (p < 0.0001).

**Figure 2** shows the cultivar difference in element uptake for B, Cu, Mn, Fe, and Zn. Unlike macroelement uptake differences between cultivars, B, Cu, Mn, Fe, and Zn, showed little variation in uptake quantities between cultivars (**Figure 2**). For minor elements, CJ2 and First Light also exhibited similar nutrient element extraction patterns when averaged across the monitored vegetative growth phase (P > 0.05) and cultivar differences in element uptake were not significantly different for most elements. However, repeated measures ANOVA revealed a significant effect of collection date (P < 0.001) for all 10 elements assessed, illustrating that uptake dynamics varied significantly throughout the 10 vegetative phase sampling intervals.

For macronutrients such as N, P, Ca, and P, a significant cultivar × date interaction was detected (P < 0.05), indicating that the cultivars responded differently to nutrient supply over time. Bonferroni’s *post hoc* comparisons showed that in early vegetative stages, CJ2 had consistently higher N and K uptake as compared to First Light, though differences declined later in the vegetative stage. For example, on July 24, N uptake for CJ2 exceeded that of First Light by 25% (P = 0.014). Linear mixed-effects models supported the stability of these findings after controlling for potential outlier influence, showing no significant differences between cultivars in total element uptake over time for Zn, Fe, Mn, and Cu, although all exhibited significant temporal variation in uptake (P < 0.001) consistent with additional extraction as biomass increased. The element uptake per unit of H_2_O transpired did provide an estimate of the daily nutrient replenishment amounts required to maintain stable nitrate and potassium concentrations in the input nutrient solution.

Mean nutrient uptake from solution for each cultivar was compared to predicted concentrations derived from leaf, stem, and root tissues, as well as organ biomass-weighted means (**Table 1**). The accuracy of each prediction varies by cultivar, element, and organ. For N, the organ-weighted mean most closely matched solution uptake in CJ2 and First Light, with deviations of +2.9% and –3.1%, respectively. Leaf tissue consistently overpredicted N uptake (34.3% in CJ2; 25.6% in First Light), while root values underpredicted uptake in both cultivars by 19.4% and 5.9%. Stem predictions were the least accurate, underestimating nutrient uptake by 36.3% (CJ2) and 37.3% (First Light). P uptake was best predicted by root tissue concentrations in both cultivars, with a small overprediction of 7.5% (CJ2) and underprediction of 20.3% (First Light). Stem P values were again least accurate, underpredicting solution uptake by 65.8% in CJ2 and 59.2% in First Light. Organ-weighted means also underpredicted P uptake by 51% (CJ2) and 46.5% (First Light). For K, solution uptake was best predicted by the root tissue element concentration in CJ2 (–26.6%). First Light stem values, on the other hand, were the closest to predicting potassium uptake (–19.7%), however, stem concentrations underpredicted K uptake in CJ2 (29.7%). Ca uptake was best approximated by the organ biomass-weighted mean in CJ2 (–15.8%) and by the leaf concentration in First Light (–4.5%), the latter representing the most accurate prediction for any tissue in that cultivar. In First Light, however, the organ biomass-weighted mean underpredicted by 27.7%, whereas in CJ2 the stem underpredicted Ca uptake by 54.7%, and leaf values overestimated uptake for CJ2. For Mg, uptake was best predicted by the leaf concentration in both First Light (–31.2%) and CJ2 (–4.7%), which more closely matched nutrient solution uptake than either stem or root estimates. In both cultivars, root predictions significantly underpredicted Mg uptake; CJ2 (42.7%) and First Light (43.1%) and stem estimates were the least accurate, deviating by –74.4% (CJ2) and –72.3% (First Light). Organ biomass-weighted means were intermediate at –38.3% and –49.8% in CJ2 and First Light, respectively.

**Table 1:** Mean boron [B], copper [Cu], calcium [Ca], iron [Fe], magnesium [Mg], manganese [Mn], nitrogen [N], phosphorus [P], potassium [K], and zinc [Zn] uptake from solution (Uptake) for cultivar CJ2 and First Light for ten repeated measures of 12 h day uptake cycles over the course of the vegetative phase.

	Uptake (ppm)	Leaf (ppm)	Stem(ppm)	Root (ppm)	Weighted (ppm)
CJ2					
N	121.52 ± 5.56	163.14 ± 10.34	77.51 ± 16.28	145.08 ± 5.50	125.08 ± 11.99
P	38.86 ± 1.89	19.84 ± 1.29	13.28 ± 1.67	35.94 ± 1.60	19.00 ± 1.62
K	157.62 ± 10.40	75.25 ± 4.00	110.83 ± 12.14	115.67 ± 11.14	94.76 ± 7.19
Ca	66.27 ± 2.36	84.04 ± 7.64	30.04 ± 7.30	35.99 ± 2.89	55.78 ± 6.84
Fe	1.58 ± 0.12	0.36 ± 0.03	0.15 ± 0.03	2.98 ± 0.24	0.58 ± 0.07
Mg	39.86 ± 2.25	37.98 ± 3.12	10.20 ± 2.02	22.83 ± 1.46	24.60 ± 2.39
Mn	0.19 ± 0.01	0.20 ± 0.02	0.23 ± 0.03	0.37 ± 0.03	0.23 ± 0.02
B	0.07 ± 0	0.13 ± 0	0.05 ± 0.01	0.05 ± 0	0.08 ± 0.01
Cu	0.11 ± 0.02	0.01 ± 0	0.03 ± 0.02	0.05 ± 0	0.02 ± 0.01
Zn	0.10 ± 0.02	0.10 ± 0.01	0.04 ± 0.01	0.10 ± 0	0.08 ± 0.01
First Light					
N	131.77 ± 5.25	165.60 ± 2.39	82.58 ± 6.89	139.53 ± 1.72	127.70 ± 2.46
P	38.40 ± 3.21	21.70 ± 0.44	15.69 ± 1.05	30.62 ± 1.39	20.56 ± 0.51
K	169.41 ± 13.30	77.74 ± 2.00	135.90 ± 9.54	105.29 ± 4.08	105.70 ± 3.28
Ca	69.89 ± 2.02	66.76 ± 1.66	40.54 ± 5.55	29.83 ± 2.11	50.50 ± 2.04
Fe	1.37 ± 0.30	0.27 ± 0.01	0.17 ± 0.02	2.85 ± 0.32	0.61 ± 0.04
Mg	41.03 ± 3.05	28.23 ± 0.57	11.36 ± 1.28	23.35 ± 0.88	20.58 ± 0.55
Mn	0.17 ± 0.02	0.27 ± 0.01	0.17 ± 0.02	0.34 ± 0.03	0.24 ± 0.01
B	0.08 ± 0	0.09 ± 0	0.06 ± 0.01	0.05 ± 0	0.07 ± 0
Cu	0.07 ± 0.04	0.01 ± 0	0.01 ± 0	0.04 ± 0	0.01 ± 0
Zn	0.07 ± 0.03	0.09 ± 0	0.04 ± 0	0.10 ± 0	0.07 ± 0

Values are the mean of four replicate plants per cultivar averaged over ten repeated 12 h day uptake cycles ± standard error of the mean. Cultivar CJ2 and First Light organ-explicit (Leaf, Stem, and Root; Equation 1) and organ biomass-weighted (Weighted; Equation 3) mass balance calculations in relation to water use efficiency dynamics.

Fe uptake was generally underpredicted by leaf, stem and organ-weighted values in both cultivars. The root mean concentrations predicted approximately twice the amount of measured uptake in both cultivars, overpredicting 88.6% (CJ2) and 108% (First Light). Stem values underpredicted Fe uptake by ~88% in both cultivars and were less accurate than leaves, whereas the organ-weighted mean value came the closest to uptake values at –63.3% and –55.5% for CJ2 and First Light (**Table 1**). For Mn, leaf values were the most accurate predictor of uptake in CJ2, with a deviation of only +5.3%, whereas the stem value was an exact match of measured uptake in First Light (0.17 ppm). The organ biomass-weighted mean overestimated uptake in CJ2 (21.1%) and First Light (41.2%), while root values in both cultivars (0.34–0.37 ppm) overpredicted by almost twice that of actual uptake from solution. For B, uptake was most accurately predicted by the organ biomass-weighted mean in CJ2 (+14.3%) and by either the leaf or organ biomass-weighted mean values in First Light (± 12.5%), whereas stem and root concentrations underestimated uptake by approximately 30% in both cultivars. Cu was generally underpredicted; however, the root values were the closest to measured uptake in CJ2 and First Light, respectively. Stem Cu concentrations were consistently low (<0.01 ppm) and did not closely align with uptake values in either cultivar. Both leaf and root calculated input values (0.10 ppm) were exact matches for Zn uptake from solution (0.10 ppm) in CJ2. In First Light, alternatively, the organ biomass-weighted mean matched the uptake value (0.07 ppm).

**Figure 3:**
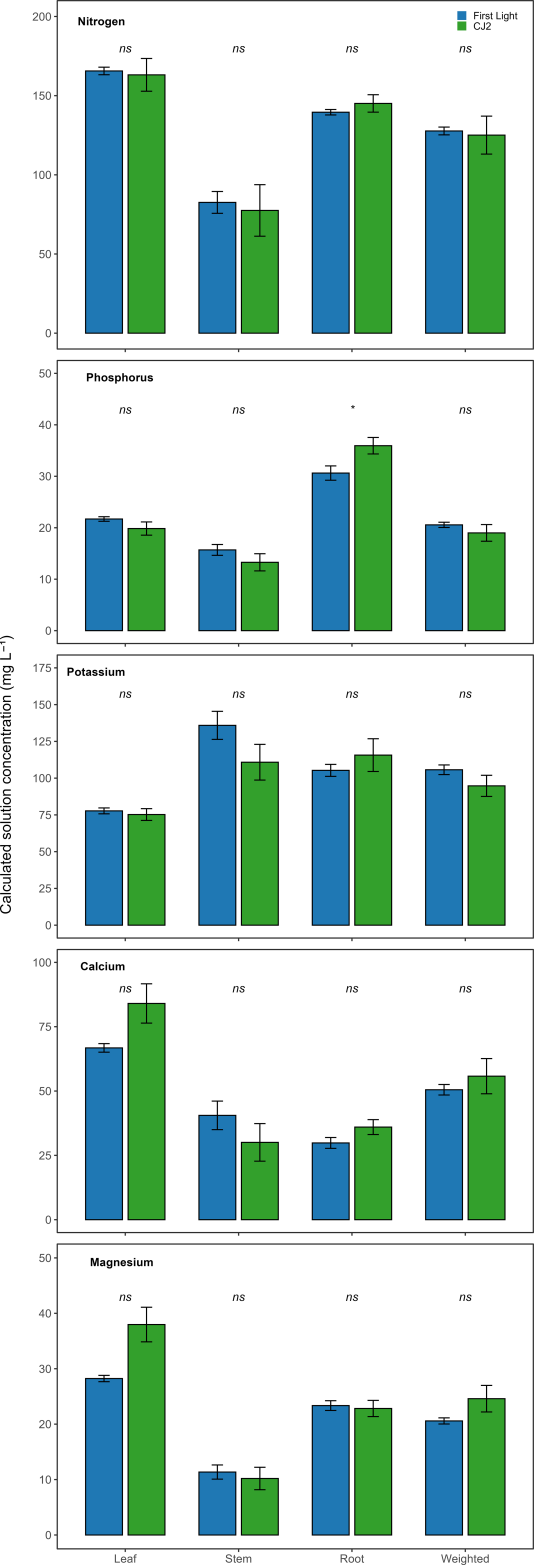
Comparison of calculated mean solution input concentrations derived from leaf, stem, root, and organ biomass-weighted tissue measurements for calcium, magnesium, nitrogen, phosphorus, and potassium in cultivars CJ2 and First Light (n = 4). Bars represent time-averaged means ± standard error across the vegetative monitoring period. “ns” denotes no significant cultivar main effect (P ≥ 0.05) based on repeated-measures ANOVA.

**Figure 4:**
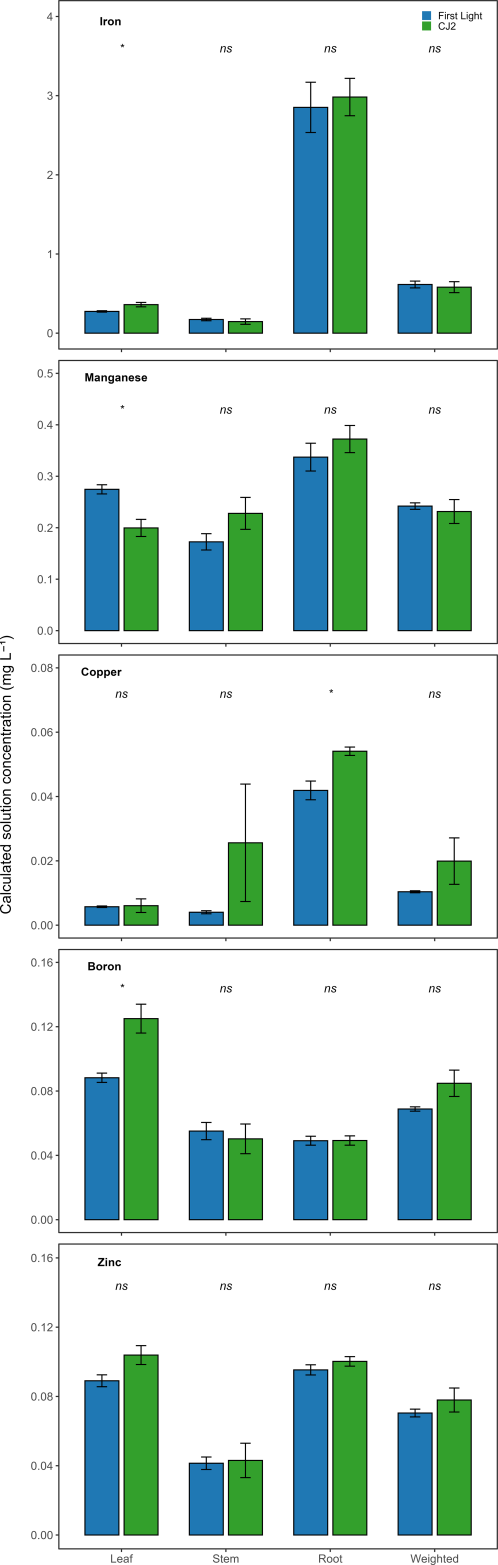
Comparison of calculated mean solution input concentrations derived from leaf, stem, root, and organ biomass-weighted tissue measurements for boron, copper, iron, manganese, and zinc in cultivars CJ2 and First Light (n = 4). Bars represent time-averaged means ± standard error across the vegetative monitoring period. “ns” denotes no significant cultivar main effect (P ≥ 0.05) based on repeated-measures ANOVA.

**Figure 3** presents cultivar comparisons of macro-element input solution concentrations calculated for each organ and as organ biomass-weighted means. With the exception of four cases, organ-specific and biomass-weighted concentrations were not significantly different. Four macro-elements exhibited cultivar-level divergence for root phosphorus, stem potassium, leaf calcium and magnesium. Nonetheless, between cultivar differences were not significant. **Figure 4** shows the corresponding micro-element comparisons. Substantial cultivar differences were observed in seven cases. These involved leaf boron, manganese, and zinc; stem and root copper; and the biomass-weighted concentrations of boron and copper.

Although mean differences between cultivars appear visually distinct for some elements and tissues in **Figures 3**, **4**, these patterns were not consistently supported by statistical separation. Apparent differences in bar height reflect numerical differences in cultivar means, whereas statistical inference additionally accounts for variability among replicate plants and the resulting uncertainty around the means. For elements such as calcium and magnesium, relatively large within-cultivar variation reduced the ability to distinguish cultivar effects despite observable mean separation. Consequently, these comparisons are interpreted as numerical trends rather than statistically robust differences. In contrast, significant cultivar differences were observed for several micronutrients. **Figure 4** illustrates were they are both visually apparent and statistically supported, highlighting that cultivar differences during the vegetative phase were element- and tissue-specific rather than uniform across nutrients.

## 4 Discussion

### 4.1 Introduction

A core principle of the mass balance framework is that absorbed elements retain their atomic identity throughout the plant’s life cycle, enabling quantitative inference of nutrient inputs from tissue composition and transpiration metrics. This biochemical constancy underpins the robustness of the mass balance approach and is consistent with established stoichiometric principles in plant systems (Balboa et al., 2018).

### 4.2 Mass balance and cultivar-specific uptake dynamics

By aligning nutrient input with water use, the mass balance framework supports biophysics-driven fertigation strategies and builds on prior work in hydroponic systems (Langenfeld et al., 2022; Westmoreland and Bugbee, 2022; Hershkowitz et al., 2025). To evaluate whether nutrient depletion from the fertigation solution reflects uptake, nutrient supply was intentionally maintained above potential uptake throughout the vegetative phase, allowing a comparison between measured nutrient uptake from solution and mass balance predictions. Observed nutrient uptake generally exceeded published sufficiency ranges, consistent with reports of luxury consumption in vegetative *C. sativa* that does not necessarily enhance yield (Saloner and Bernstein, 2020, SalonerBernstein, 2021; Shiponi and Bernstein, 2021; Westmoreland and Bugbee, 2022). Despite similar WUE between cultivars CJ2 (4.71 g L^-1^ H_2_O) and First Light (4.59 g L^-1^ H_2_O), distinct uptake patterns emerged during early growth. CJ2 exhibited significantly greater N and K uptake in the first week (**Figure 1**), with N uptake exceeding that of First Light by 25% on day of year 206 (July 24) (P = 0.014), consistent with genotypic variation in root development, nutrient transport efficiency, and internal demand (Marschner, 2012; Xu et al., 2012). Repeated measures ANOVA confirmed significant cultivar × date interactions for N, P, K, and Ca (P < 0.05), but not for Fe, Mn, Cu, or Zn, whose uptake patterns converged across cultivars over time (**Figure 2**). These results indicate that macronutrient demand is more genotype-dependent, while micronutrient uptake is increasingly driven by biomass accumulation and transpiration-mediated mass flow in later stages (Bugbee, 2004).

### 4.3 Organ-specific versus whole-plant nutrient estimates

Across all elements measured in this study, no single tissue consistently provided the best prediction of nutrient uptake from solution in either cultivar. The organ biomass-weighted mean was the most accurate predictor for N (both cultivars), Ca (CJ2), B (CJ2), and Zn (First Light). Leaf tissue most accurately predicted uptake of Mg (CJ2), Mn (CJ2), and Zn (CJ2). Root tissue concentrations were the best predictor for uptake of P (both cultivars), K (CJ2), Cu (both cultivars), and Zn (CJ2), while also providing the second-best estimates in multiple instances. Leaf values were the best predictors for Ca (First Light) and stem values were the best predictors for Mn (First Light). These findings demonstrate that while individual organ means can perform well, element and cultivar-specific patterns exist, and root or even stem concentrations can sometimes be the most accurate predictors as compared to leaf concentrations (He et al., 2015; Sperling et al., 2020). Therefore, multi-organ assessment remains valuable when precise nutrient demand estimation is required for optimizing fertigation strategies.

This organ-specific variability aligns with prior work showing that reliance on leaf tissue analysis alone can misrepresent whole-plant nutritional status, particularly for mobile macronutrients such as N, K, and Mg. Redistribution of these nutrients among organs is well documented, especially when plants mobilize resources toward developing tissues (Ingestad, 1982; Juarez-Maldonado et al., 2017). Recent meta-analyses confirm that integrating nutrient concentrations across organs, weighted by their respective biomass contribution to the whole-plant, yields a more accurate representation of whole-plant nutrient status than single-organ measurements (Sperling et al., 2020). These results demonstrate that organ biomass-weighted means, in combination with targeted organ measurements where appropriate, offer the most robust approach for inferring whole-plant nutrient uptake in mass-balance fertigation models.

Our findings reflect this complexity. The organ biomass-weighted mean consistently provided the most accurate predictions for N, Ca, B, and Zn uptake, closely matching measured solution depletion values across both cultivars. For P, K, and Cu, root concentrations provided the best match, while leaf concentrations were most accurate for Mg in certain cases. No single organ type consistently predicted element uptake for all elements or cultivars, underscoring the element- and genotype-specific nature of nutrient partitioning. Importantly, this kind of discrepancy between tissue-derived estimates and solution-based uptake has also been observed in other substrate-grown crops. In tomato grown in free-draining perlite, Cedeño et al. (2024) reported that K uptake calculated from plant dry matter consistently underestimated K consumption measured by the nutrient balance method, by approximately 14–46%, largely due to retention and loss processes occurring within the cropping system rather than the plant itself (Cedeño et al., 2024). This parallels the underprediction of K uptake in our study and may indicate that system-level retention, rather than biological inconsistency, contributes to the mismatch between measured solution depletion and tissue-based estimates. Therefore, estimates derived from the mass balance approach are imperfect in their current form, as evidenced by the underprediction of K uptake under the conditions tested. Rather than introducing *ad hoc* compensation parameters, we treat these discrepancies as informative targets for future investigation.

### 4.4 Broader implications and operational considerations

While the static measurement of WUE and tissue concentrations in this study captures a snapshot of nutrient–water dynamics, it lacks the temporal resolution necessary to capture shifting nutrient demand throughout the vegetative growth stage. Prior studies have demonstrated that seasonal and ontogenetic changes can substantially alter nutrient uptake patterns, indicating that repeated biomass and transpiration measurements would improve model fidelity (Juarez-Maldonado et al., 2017). Implementing mass balance strategies also requires precise quantification of water use and organ-level nutrient accumulation, more feasible in controlled environments than in open-field systems. However, the increasing use of closed-loop systems with real-time monitoring capabilities provides an ideal platform for adaptive fertigation management (Houston, 2021).

The mass balance framework applied here does not explicitly resolve biological complexities that regulate nutrient acquisition, including ion antagonism, competitive uptake among chemically similar elements, and transporter-level control that can modify uptake efficiency independently of external nutrient availability. In addition, internal regulatory processes such as nutrient remobilization, storage, and translocation among organs may decouple instantaneous uptake from tissue accumulation, particularly for mobile elements. Accordingly, the uptake estimates reported in this study should be interpreted as condition-specific observations under non-limiting nutrient supply rather than prescriptions for fertilization optimization. Addressing these physiological and regulatory processes will be necessary in future work to more fully integrate mass balance approaches with plant nutritional demand and to improve their applicability for nutrient management. Nonetheless, the mass balance approach presents a compelling alternative to fixed nutrient recipes by enabling feedback-driven fertigation protocols. This is particularly advantageous in high-value crops such as *C. sativa*, where genotype-specific nutrient demands are pronounced. In semi-closed or zero-discharge systems, these approaches can minimize nutrient loss and environmental impact by more closely aligning supply with uptake.

## 5 Conclusions

This study provides preliminary evidence that a mass-balance framework can estimate nutrient uptake in *C. sativa*. By integrating transpiration-driven water flux with tissue nutrient concentrations, we derived empirically supported estimates of nutrient uptake per unit of water transpired. This integration allows irrigation nutrient solution input concentrations to be estimated to correspond with plant uptake. Critically, the results also highlight the limitations of relying solely on single-organ nutrient concentrations, particularly leaf tissue, for predicting whole-plant nutrient status. Given the substantial differences in elemental concentrations among roots, stems, and leaves, an organ-inclusive, biomass-weighted approach provides a more accurate whole-plant estimate (He et al., 2015; Tang et al., 2018; Jing et al., 2020; Jiang et al., 2024).

By linking nutrient inputs to transpiration and tissue accumulation, this framework facilitates closed-loop feedback control, enabling fertigation to be dynamically adjusted in response to actual plant uptake. Such adaptive protocols enhance resource use efficiency, reduce nutrient waste, and minimize environmental impact, particularly in recirculating hydroponic and semi-closed systems (Houston, 2021; Langenfeld et al., 2022).

## Data Availability

The raw data supporting the conclusions of this article will be made available by the authors, without undue reservation.
